# Oxygen Depletion in FLASH Particle Therapy: Effects of Linear Energy Transfer and Ion Track Structure

**DOI:** 10.3390/antiox15030331

**Published:** 2026-03-06

**Authors:** Jintana Meesungnoen, Jean-Paul Jay-Gerin

**Affiliations:** Department of Medical Imaging and Radiation Sciences, Faculty of Medicine and Health Sciences, Université de Sherbrooke, 3001, 12th Avenue Nord, Sherbrooke, QC J1H 5N4, Canada; jintana.meesungnoen@usherbrooke.ca

**Keywords:** FLASH radiotherapy, cellular water radiolysis, Monte Carlo multi-track chemical modeling, radiolytic oxygen depletion (consumption) (ROD), linear energy transfer (LET), radiation track structure, chemical yields (*G* values), hadrontherapy

## Abstract

Ultra-high dose-rate (FLASH) irradiation can transiently deplete oxygen and modulate radical-mediated chemistry in irradiated cells. Cellular antioxidants also contribute to mitigating oxidative damage in a manner dependent on linear energy transfer (LET), as suggested by recent experimental studies. In this work, we employed our multi-track Monte Carlo simulation framework (IONLYS-IRT) to investigate how LET influences transient radiation-induced oxygen depletion (ROD) in a cell-like aqueous environment under FLASH irradiation conditions. FLASH exposures were modeled as single, instantaneous pulses of protons with energies from 300 MeV to 150 keV, corresponding to LET values of ~0.3 to 71 keV/μm. Our simulations revealed a marked decline in oxygen depletion with increasing LET, in agreement with experimental observations. For an intracellular O_2_ concentration of 30 μM, the oxygen consumption yield, *G*(–O_2_), decreased from ~4.0 molecules/100 eV at low LET (~0.3 keV/μm) to ~1.6 molecules/100 eV at high LET (~71 keV/μm), representing a ~60% reduction. To assess whether ROD depends solely on LET or is also governed by ion track structure, we systematically compared multiple ion species (protons, ^4^He^2+^, ^10^B^5+^, ^12^C^6+^, ^16^O^8+^, ^20^Ne^10+^, ^28^Si^14+^, ^32^S^16+^, and ^40^Ar^18+^) at comparable LET values. At ~70 keV/μm, heavier ions produced significantly higher *G*(−O_2_) values than protons—though still below those at low LET—suggesting that track structure plays a key role beyond LET alone. These findings highlight the dual importance of LET and ion-specific track structure in modulating ROD under FLASH conditions. Notably, enhanced ROD in surrounding normal tissues (low-LET plateau regions) may contribute to radioprotective effects, whereas reduced ROD in tumor tissues (high-LET Bragg peak regions) would be expected to preserve tumoricidal efficacy. Together, these results provide a mechanistic framework for optimizing proton and heavy-ion approaches in FLASH radiotherapy.

## 1. Introduction

Radiation therapy (RT) is a cornerstone of modern cancer treatment, used alongside surgery, chemotherapy, and immunotherapy. By delivering ionizing radiation to eradicate malignant cells, RT plays a central role in standard oncologic care [1]. Major technological advances, including intensity-modulated, stereotactic, spatially fractionated, and image-guided techniques, have greatly enhanced dose conformity, delivery precision, and normal-tissue sparing. Despite these improvements, a persistent challenge remains: delivering tumoricidal radiation doses while minimizing both acute and late toxicities to surrounding healthy tissues.

Over the past decade, this challenge has spurred growing interest in ultra-high-dose-rate irradiation, known as FLASH radiotherapy (FLASH-RT) [2,3], which delivers radiation at mean dose rates exceeding ~40–150 gray per second (Gy/s)—several thousand times higher than those used in conventional (CONV) clinical radiotherapy. Numerous preclinical studies, conducted in vitro and in vivo using cell cultures, organ systems, and experimental animal models, have shown that FLASH-RT can markedly spare normal tissues while maintaining tumor control comparable to that achieved with CONV dose-rate RT (for reviews, see [4,5,6,7,8,9]). These observations challenge long-standing principles of classical radiobiology [10,11,12].

Initially observed using electron beams with instantaneous dose rates on the order of 10^6^–10^7^ Gy/s, the FLASH effect has since been demonstrated with megavoltage photons, protons, helium, and heavier ions (see, e.g., [8,13,14,15,16,17,18]), substantially expanding its potential clinical relevance. Early clinical experiences, including first-in-human treatments with electron and proton FLASH [19,20,21], have further supported its feasibility and therapeutic promise.

Despite the rapid accumulation of experimental and clinical evidence, the physicochemical and biological mechanisms underlying the normal-tissue sparing observed with FLASH-RT remain poorly understood. Several potentially interconnected hypotheses have been proposed, including transient oxygen depletion, alterations in reactive oxygen species (ROS) and free-radical chemistry, modulation of DNA damage induction and repair, mitochondrial responses, and immune regulation (see, e.g., [5,6,7,9,22,23,24,25,26,27,28,29,30,31,32,33,34,35,36,37,38,39,40,41]). Among these, the transient radiation-induced oxygen depletion (TOD or ROD, depending on the authors) hypothesis is the most widely discussed and is regarded by many as a leading explanation for the FLASH effect. It postulates that oxygen is depleted by FLASH irradiation faster than it can be replenished, such that the rapid radiolytic consumption of oxygen during ultra-short irradiation pulses induces a transient hypoxic state in initially well-oxygenated normal tissues, thereby temporarily reducing radiosensitivity [42]. While supported by early experimental studies, the extent to which oxygen depletion alone can account for the FLASH effect in model solutions and in vivo remains actively debated, as recent findings suggest that ROD, by itself, may be insufficient to generate the degree of hypoxia required to fully explain normal-tissue sparing, thereby pointing to the involvement of additional complementary mechanisms or modulating factors [41,43,44,45,46].

In a previous study [47], we examined the ROD hypothesis from a radiation-chemical perspective using a computational model to investigate dose-rate effects on water radiolysis in an aqueous environment mimicking a confined cellular space exposed to instantaneous 300-MeV proton (LET ~0.3 keV/μm) pulses. Simulations were performed with our Monte Carlo multi-track chemistry code IONLYS-IRT [48,49], optimized for a homogeneous, aerated medium (30 μM O_2_) containing water, carbon-based biomolecules (RH), radiation-induced radicals (R^•^), and major cellular antioxidants, including glutathione (GSH), ascorbate (AH^−^), nitric oxide (^•^NO), and α-tocopherol (TOH). The model tracked the temporal evolution of these species, with particular emphasis on oxygen consumption, from the picosecond to the second time scale. The results suggested that transient oxygen depletion plays a significant role in normal-tissue sparing under FLASH irradiation in vivo. Nevertheless, under typical FLASH conditions with a ~30 Gy dose delivered at instantaneous dose rates of the order of 10^6^–10^7^ Gy/s, simulations showed that only ~45% of intracellular O_2_ is consumed, indicating that oxygen depletion alone can account for only part of the observed FLASH effect.

Following the publication of this work, we investigated whether variations in the initial oxygen concentration would affect the extent of ROD under otherwise identical irradiation conditions. This analysis aimed to evaluate the sensitivity of our results to deviations from the assumption of cellular homogeneity inherent in our simulations. We found that the predicted oxygen depletion of ~45% at a dissolved O_2_ concentration of 30 μM increased to nearly 65% when the initial concentration was reduced to 20 μM, and approached complete depletion (~100%) at 10 μM O_2_. These results are particularly noteworthy, as they suggest that even modest variations in baseline oxygenation could significantly impact cellular responses during FLASH irradiation, with poorly oxygenated regions at heightened risk of undergoing near-complete oxygen depletion.

Based on these results, which align well with available experimental observations, and without presuming the possible involvement of additional mechanisms or modulatory factors, it can be reasonably argued that the ROD hypothesis captures, to a large extent, the essential physicochemical features of the FLASH effect, particularly its strong dependence on local oxygen availability (for a comprehensive review on the oxygen puzzle in FLASH-RT, see [5]).

Beyond oxygen depletion alone, it is of particular interest to examine whether the ROD framework can also explain the recently observed dependence of oxygen consumption on linear energy transfer (LET). Specifically, experimental results have shown a marked decrease in oxygen consumption with increasing LET [16]. Investigating the LET dependence of FLASH-RT is a relatively recent development, as most studies so far have used radiation sources with relatively low LET values (see, e.g., [5]), despite the wide range of biological models employed. The study by Karle et al. [16], which extended the LET range up to 65 keV/μm and 100.3 keV/μm using carbon (^12^C) and oxygen (^16^O) ion beams, respectively, provides key insights into this question. While ROD may not be sufficient to fully account for the entire FLASH effect, it could nonetheless play a significant role in mediating the LET-dependent responses observed experimentally. Exploring this possibility is the central objective of the present study.

## 2. Materials and Methods

This study builds on our previously reported radiation-chemical modeling framework using our Monte Carlo multi-track chemistry simulation code IONLYS-IRT [47,48,49]. The cellular water model and reaction scheme employed here are identical to those described in our earlier work [47]. For completeness, a brief summary is provided below. Full methodological details are available in references [47,48].

### 2.1. The ‘Instantaneous Pulse’ (Dirac) Model for FLASH Radiolysis

In our previous work [48], we investigated the radiolysis of water and aqueous solutions using single, instantaneous pulses of *N* incident 300-MeV protons. These protons produce a low LET of ~0.3 keV/μm, comparable to that of Compton electrons generated by ^60^Co γ-rays or other fast (MeV) electrons (see, e.g., [50,51]). Owing to this low LET, their initial track structure consists of small, well-separated Magee-type spurs—localized, quasi-spherical clusters of radiolytic species distributed along each proton path [52,53,54].

In our model, monoenergetic protons are assumed to strike the water surface perpendicularly over a circular area of radius *R*_o_ (set to 0.1 μm; see Figure 1 in [48]). Under the ‘instantaneous pulse’ (Dirac) approximation [55], the pulse duration is considered to be zero, resulting in the simultaneous formation of all primary radiolytic species. Given their essentially rectilinear trajectories, the irradiation geometry is modeled as a cylindrical volume, with *N* proton tracks aligned along the cylinder axis and interacting over a defined track length. This configuration closely resembles that used in our earlier Monte Carlo simulations at low dose rates [49], except that here, multiple interacting tracks (*N*) are simulated simultaneously within a single pulse, rather than a single isolated track. The corresponding proton fluence is given by *N*/π*R*_o_^2^.

In our more recent study [47], we explored dose-rate effects by varying *N* from 1 (representing a single isolated track with no dose-rate effect) to 50, while keeping the proton energy—and therefore the LET—constant. As previously established (see Figure 3B in [48]), an *N* value of 20 corresponds to an instantaneous absorbed dose rate of ~10^6^–10^7^ Gy/s, which aligns with the dose-rate range commonly associated with FLASH irradiation in current experimental studies.

“Time zero” is defined as the moment the proton pulse reaches the entrance of the irradiated volume.

### 2.2. Monte Carlo Multi-Track Chemistry Simulations: Predicting Chemical Yields (G Values)

We conducted this study using our IONLYS-IRT Monte Carlo code to simulate the radiolysis of our previously developed aerated cellular water system under FLASH irradiation conditions [47] (see infra).

Briefly, the event-by-event IONLYS program simulates the physical and physicochemical stages of radiation action in a three-dimensional environment, tracking energy deposition and spur formation up to ~1 picosecond (ps). It explicitly models the conversion of local physical products into the initial radical and molecular radiolysis species, including the hydrated electron (e^−^_aq_), hydrogen atom (H^•^), molecular hydrogen (H_2_), hydroxyl radical (^•^OH), hydrogen peroxide (H_2_O_2_), hydronium ion (H^+^), and hydroxide ion (OH^−^), with H^+^, e^−^_aq_, and ^•^OH being produced in the highest concentrations [49,53,54,56]. The resulting highly nonhomogeneous spatial distribution of reactive species serves as the starting point for the subsequent chemical stage of radiolysis, which begins beyond ~1 ps.

The chemical stage is simulated using our IRT program [49,57], which implements the “independent reaction times” (IRT) method [58,59] to model the random diffusion and mutual or solute-mediated reactions of radiolytic species—without explicitly tracking individual diffusion trajectories. The accuracy of this approach in predicting chemical yields (*G* values) has been validated across a wide range of irradiation conditions through comparisons with step-by-step Monte Carlo simulations (see, e.g., [60,61]). The IRT method is particularly well suited for modeling longer time scales, during which reactive species become uniformly distributed throughout the solution.

Throughout this work, *G* values are expressed as the number of molecules formed or consumed per 100 eV of absorbed energy. For conversion to SI units, note that 1 molecule per 100 eV ≈ 0.103364 μmol/J [54].

### 2.3. Water Radiolysis in Our Cell Model: Chemical Reaction Scheme

In this study, the cell is modeled as a homogeneous aqueous medium, thereby neglecting the complexity of intracellular heterogeneity. Real cells consist of multiple compartments—such as the cytoplasm, nucleus, mitochondria, and membranes—each with distinct chemical and physical properties. Despite this simplification, assuming homogeneous kinetics provides a practical and insightful framework for identifying the dominant reactive species and reaction pathways under high-intensity pulsed irradiation conditions [62,63,64]. Within this context, our simplified cellular water model proves especially effective for elucidating the primary chemical mechanisms associated with high dose-rate irradiation and the FLASH effect.

The model includes dissolved oxygen at a concentration of 30 μM, representative of well-oxygenated mammalian tissues in vivo (see, e.g., [65,66]). It also incorporates a generic pool of oxidizable, carbon-based biomolecular targets—collectively treated as a single RH species—which encompasses proteins, nucleic acids (DNA and RNA), free amino acids and nucleotides, and membrane lipids. The concentration of RH is set to 1 M, consistent with previous modeling studies [36,67,68]. Under the FLASH irradiation conditions considered here, the initial concentration of bio-radicals (R^•^) formed via the direct ionization of RH, followed by deprotonation of RH^•+^, is estimated to be ~2.5 μM, based on previously reported direct-action yields [23,36,69].

As mentioned above, the cellular antioxidants considered in this study include glutathione, ascorbate, nitric oxide, and α-tocopherol. The concentrations used—6.5 mM GSH, 1 mM AH^−^, 1 μM ^•^NO, and 0.2 mM TOH—are based on our previous study [47], which also provides a detailed account of their respective roles and reaction pathways within the irradiated cellular water model being studied. These values reflect typical intracellular concentrations reported for well-oxygenated mammalian cells and are consistent with estimates found in the literature under both physiological and irradiation conditions.

In brief, the standard IONLYS-IRT reaction scheme for the radiolysis of pure water at 25 °C under 300-MeV proton irradiation was extended in [47] to include 48 additional reactions relevant to a cell-like aqueous environment containing 30 μM dissolved oxygen. These reactions, along with their corresponding rate constants and data sources, are fully reported in Table 1 of [47] and are not reproduced here in order to maintain clarity and focus on the new developments presented in the current study.

### 2.4. Radiation-Induced Oxygen Depletion (ROD): Effects of LET and Ion Track Structure

The primary objective of this study is to evaluate the validity and limitations of the transient ROD hypothesis as a mechanistic explanation for the FLASH effect under varying radiation qualities—specifically, as a function of LET and track structure.

To this end, we first employed our multi-track Monte Carlo simulation framework to model FLASH exposures using single, instantaneous pulses of protons with energies ranging from 300 MeV down to 0.15 MeV. This energy range corresponds to LET values between ~0.3 and 71 keV/μm [50,70]. In all simulations, the number of protons per pulse was fixed at *N* = 20, as previously described, to represent typical FLASH irradiation conditions.

In the second part of the study, we investigated whether ion track structure exerts an influence on ROD beyond what is predicted by LET alone. It is well established that LET is not a unique parameter to fully describe the radiation chemical effects associated with heavy-ion tracks (see, e.g., [49,71]). Accurately predicting the effects of radiation type and energy in radiolysis requires detailed knowledge of the initial spatial distribution of radiation-induced species, which reflects the microscopic energy deposition patterns unique to each ion. To explore this effect, we simulated irradiations using several ion species—protons, ^4^He^2+^, ^10^B^5+^, ^12^C^6+^, ^16^O^8+^, ^20^Ne^10+^, ^28^Si^14+^, ^32^S^16+^, and ^40^Ar^18+^—all under identical LET conditions (~70 keV/μm). This approach allows us to assess whether variations in track structure produce distinguishable effects on ROD.

Finally, we compared our simulated oxygen consumption yields in our modeled bulk cellular water system with the recent experimental measurements by Karle et al. [16], who quantified radiolytic oxygen consumption rates in 5% bovine serum albumin (BSA) solutions subjected to FLASH irradiation. Their study, which employed protons, helium, carbon, and oxygen ions over a wide LET range (5.4 to 100.3 keV/μm), provides the first experimental dataset suitable for benchmarking the LET dependence of ROD in biological relevant systems.

## 3. Results and Discussion

We present below the results of our Monte Carlo simulations assessing the influence of LET and track structure on transient oxygen depletion under FLASH irradiation conditions. These findings are subsequently discussed in terms of their underlying physicochemical mechanisms and potential therapeutic relevance. Unless otherwise stated, all results pertain to our modeled cell-like aqueous system containing 30 μM dissolved oxygen at 25 °C, as previously described [47].

**Figure 1:**
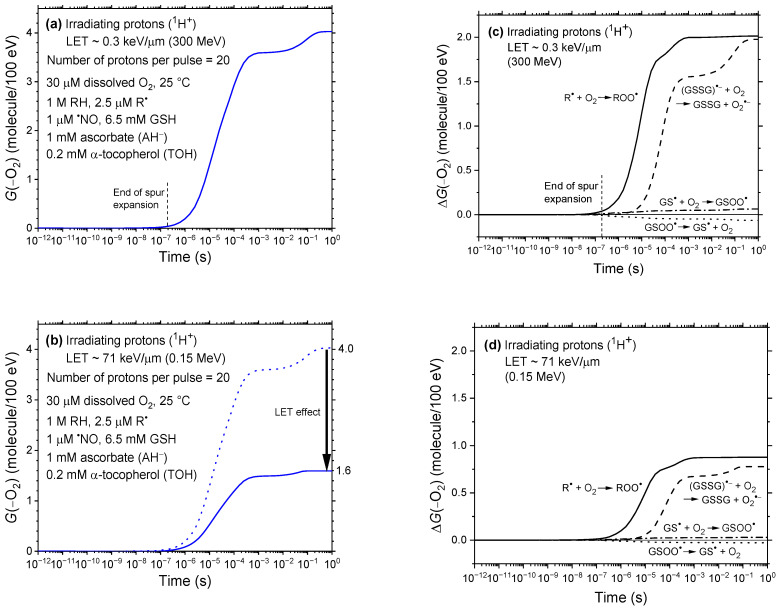
Panels (**a**,**b**): Time evolution of the oxygen consumption yield, *G*(–O_2_), calculated using our IONLYS-IRT Monte Carlo track-chemistry simulations for the radiolysis of our modeled cell-like aqueous system containing 30 μM dissolved O_2_ at 25 °C. The simulations span the timescale from 1 ps to 1 s and involve *N* = 20 incident protons per pulse at energies of 300 and 0.15 MeV, corresponding to LET values of ~0.3 and 71 keV/μm, respectively. Panel (**b**) highlights the inverse relationship between LET and *G*(–O_2_), which drops from ~4.0 to 1.6 molecules/100 eV as LET rises from ~0.3 to 71 keV/μm. Panel (**c**,**d**): Time-dependent extents Δ*G*(–O_2_), expressed in molecules per 100 eV, for the individual reactions contributing to *G*(–O_2_) over the same time range (1 ps to 1 s), based on the same simulations. Oxygen consumption arises primarily from Reactions (1)–(4), involving R^•^ and GSSG^•−^ radicals. The vertical dashed line at ~0.2 μs in Panels (**a**,**c**) marks the end of spur expansion, i.e., the transition from nonhomogeneous to homogeneous kinetics in the absence of dose-rate effects.

Figure 1a,b show the time evolution of the oxygen consumption yield, *G*(–O_2_), in our irradiated cellular water model from 1 ps to 1 s, for incident protons of 300 MeV and 0.15 MeV, corresponding to LET values of ~0.3 and 71 keV/μm, respectively. Figure 1c,d provides a detailed breakdown of the time-dependent contributions, Δ*G*(−O_2_), of individual reactions to the total oxygen depletion, as determined by our IONLYS-IRT Monte Carlo simulations. For reference, the Δ*G*(–O_2_) profile for low-LET 300 MeV protons—previously reported in Figure 3b of [47]—is reproduced in Figure 1c to allow direct comparison with the corresponding high-LET case shown in Figure 1d for 0.15 MeV protons.

As a brief reminder, our simulations revealed that dissolved oxygen is transiently depleted primarily through reactions with bio-radicals (R^•^), leading to the formation of peroxyl radicals (ROO^•^):

(1) R^•^ + O_2_ ⟶ ROO^•^ (*k* ~ 2 × 10^9^ M^−1^ s^−1^)

and, to a comparable extent, from reactions involving glutathione and glutathione disulfide radical anions (GSSG^•−^), proceeding via [47]:

(2) ^•^OH + GSH ⟶ GS^•^ + H_2_O (*k* ~ 1.4 × 10^10^ M^−1^ s^−1^)

(3) GS^•^ + GSH ⟶ GSSG^•−^ + H^+^ (*k* ~ 3.5 × 10^8^ M^−1^ s^−1^)

(4) GSSG^•−^ + O_2_ ⟶ GSSG + O_2_^•−^ (*k* ~ 5.1 × 10^8^ M^−1^ s^−1^)

Through these pathways, *G*(–O_2_) rises rapidly once chemical homogeneity is reached—typically after the microsecond timescale—and plateaus at ~4.0 molecules/100 eV by 1 s at low LET (Figure 1a). In contrast, it reaches only ~1.6 molecules/100 eV at 71 keV/μm (Figure 1b). This pronounced reduction in *G*(–O_2_) as proton LET increases from ~0.3 to 71 keV/μm highlights the strong dependence of radiation-induced oxygen depletion on LET.

What mechanisms underlie the observed decrease in *G*(–O_2_) with increasing LET? The explanation lies in fundamental principles of radiation chemistry. As shown in Figure 1c,d and Reactions (1)–(4), the extent of oxygen depletion depends on the availability of reactive radicals—primarily R^•^ and GSSG^•−^—that react with molecular oxygen. The greater the number of radicals formed, the higher the potential for oxygen consumption. Such a radical-rich environment is favored at low LET, where the radiation track structure initially consists of sparsely distributed, quasi-spherical spurs [52,53,54]. These well-separated spurs minimize early radical–radical recombination and enhance net radical production, thereby promoting efficient O_2_ scavenging. As a result, *G*(–O_2_) reaches its highest values under these conditions.

**Figure 2:**
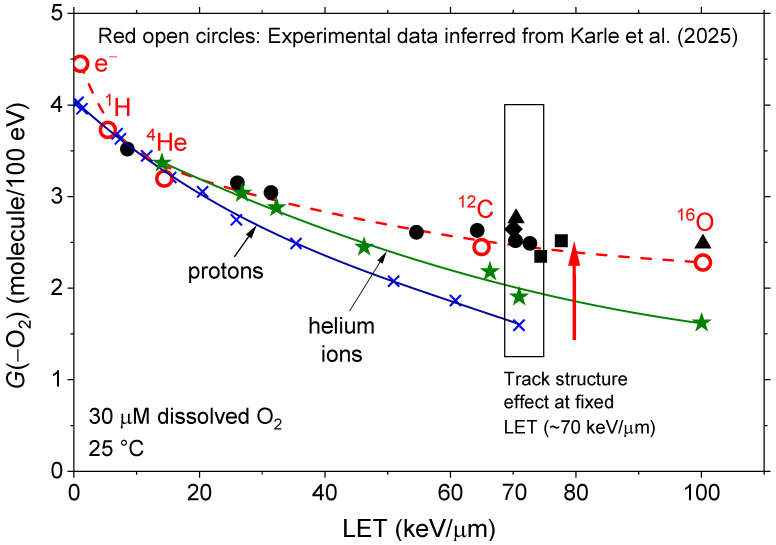
LET dependence of the oxygen consumption yield, *G*(–O_2_), at ~1 s, as obtained from our IONLYS-IRT Monte Carlo multi-track simulations of radiolysis in a modeled cell-like aqueous system containing 30 μM dissolved O_2_ at 25 °C, under identical irradiation conditions (*N* = 20 incident ions per pulse). The blue solid line and associated (×) data points show calculated *G*(–O_2_) values for incident protons with energies between 300 and 0.15 MeV (LET ~ 0.3–71 keV/μm), whereas the green solid line and associated data points (represented by a star) show the corresponding results for ^4^He^2+^ ions up to LET ~ 100 keV/μm. Additional symbols denote simulated *G*(–O_2_) values for various ion species, including ^12^C^6+^ (●), ^16^O^8+^ (▲), ^20^Ne^10+^ (◆), and ^40^Ar^18+^ (■). Red open circles represent the experimental data of Karle et al. [16], reporting oxygen consumption rates (in mmHg/Gy) for electrons, protons, helium, carbon, and oxygen ions. These rates were converted to *G*(–O_2_) values (in molecules/100 eV) using calibration to our simulated proton results at LET ~5.4 keV/μm (see text). The vertical rectangle at LET ~70 keV/μm highlights the pronounced ion track-structure effect, further analyzed in Figure 3. The red dashed line is drawn through Karle et al.’s experimental data points to guide the eye. Notably, the authors [16] did not specify the charge state of the ions used, only their LET values.

In contrast, at high LET, the spacing between adjacent spurs becomes so small that they effectively merge into a dense, quasi-continuous cylindrical track along the ion’s trajectory [49,71]. Under these conditions, the early stages of radiolysis are characterized by high local concentrations of reactive species, greatly increasing the likelihood of radical–radical recombination before these species can react with O_2_. As a result, molecular products are preferentially formed at the expense of radicals [53,72], leading to a substantial reduction in oxygen consumption via Reactions (1)–(4). This mechanism not only accounts for the strong inverse correlation between LET and *G*(–O_2_) observed in our simulations, but also aligns closely with the recent experimental findings of Karle et al. [16], involving ion beams spanning a broad LET range (5.4–100.3 keV/μm), as illustrated in Figure 2.

Figure 2 summarizes the central findings of this study. It clearly shows the monotonic decrease in simulated oxygen consumption yields as a function of LET for pulsed proton irradiations in our modeled cellular water. Specifically, *G*(–O_2_) decreases from ~4.0 molecules/100 eV at low LET (~0.3 keV/μm) to ~1.6 molecules/100 eV at high LET (~71 keV/μm), representing a nearly 60% reduction as the LET approaches the Bragg peak region (see, e.g., [49]).

Although no experimental measurements with pulsed protons at such LETs are available for our system, the oxygen consumption data reported by Karle et al. [16] offer a valuable benchmark for comparison. To enable this, we normalized their measured oxygen consumption rate in 5% BSA solutions exposed to pulsed protons at 5.4 keV/μm (0.294 ± 0.005 mmHg/Gy) to our simulated *G*(–O_2_) value at the same LET (~3.73 molecules/100 eV). This calibration enabled us to infer corresponding *G*(–O_2_) values for helium (14.4 keV/μm), carbon (65 keV/μm), and oxygen (100.3 keV/μm) ions using the experimental data in Table 3 of [16]. These inferred values are plotted in Figure 2, allowing direct quantitative comparison with our simulation results.

The agreement is remarkable: our calculated *G*(–O_2_) values—~3.36 molecules/100 eV for ^4^He^2+^ at ~14 keV/μm, ~2.63 for ^12^C^6+^ at ~64.3 keV/μm, and ~2.48 for ^16^O^8+^ at ~100.3 keV/μm—closely match the experimental values inferred from the data of Karle et al. [16]. Overall, the results presented in Figure 2 provide strong, independent support for the predicted LET-dependent decline in ROD, thereby reinforcing the robustness of our simulation approach.

While the overall agreement across ion types in the LET range of ~0.3 to 100.3 keV/μm is compelling, a marked discrepancy emerges when comparing *G*(–O_2_) values for protons versus heavier ions at similar LETs. As shown in Figure 2, this divergence becomes increasingly pronounced with rising LET. For instance, at ~70 keV/μm—where the difference is greatest—*G*(–O_2_) drops to ~1.6 molecules/100 eV for protons but rises to ~2.5 molecules/100 eV for ^12^C^6+^ ions. This finding suggests that LET alone is insufficient to fully characterize radiolytic oxygen consumption, a result consistent with Bethe’s theory of stopping power [73]. Instead, it points to a distinct track structure effect [49,71,74]—rooted in the ion-specific spatial distribution of energy deposition events—that critically influences the chemistry of irradiated systems. Understanding this effect thus appears essential for interpreting LET-dependent outcomes in FLASH irradiation and hadron therapy.

This dependence of *G*(–O_2_) on the ion type is illustrated in Figure 2 under identical irradiation conditions at a fixed LET of ~70 keV/μm. As shown, the *G*(–O_2_) values for protons (~1.6 molecules/100 eV) and helium ions (~1.9 molecules/100 eV) are the lowest and closely similar. In contrast, heavier ions—^12^C^6+^ (~2.54), ^16^O^8+^ (~2.76), ^20^Ne^10+^ (~2.64), and ^40^Ar^18+^ (~2.43 molecules/100 eV)—exhibit significantly higher yields that tend to cluster together. These results, highlighted by the vertical rectangle centered around 70 keV/μm in Figure 2, underscore the influence of ion track structure. Nevertheless, all values remain well below the ~4.0 molecules/100 eV observed for low-LET 300 MeV protons.

**Figure 3:**
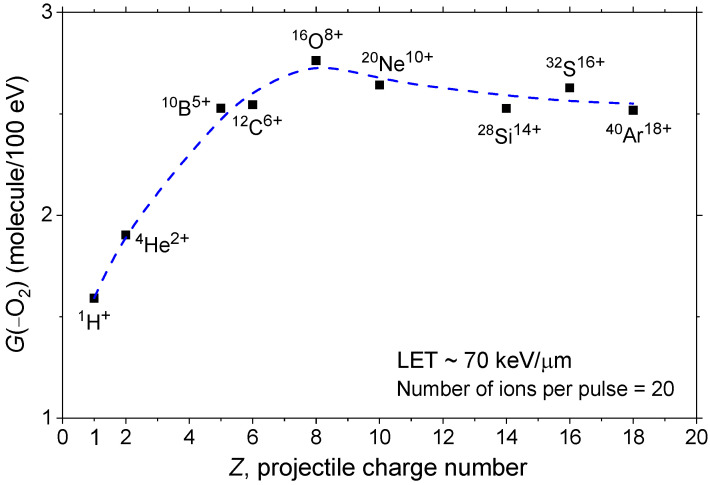
Oxygen consumption yield, *G*(–O_2_), at ~1 s, as a function of ion charge number (Z), obtained from our IONLYS-IRT Monte Carlo simulations of radiolysis in our modeled cell-like aqueous system containing 30 μM dissolved O_2_ at 25 °C. Simulations were performed for ^1^H^+^, ^4^He^2+^, ^10^B^5+^, ^12^C^6+^, ^16^O^8+^, ^20^Ne^10+^, ^28^Si^14+^, ^32^S^16+^, and ^40^Ar^18+^ ions, all at an LET of ~70 keV/μm, under identical irradiation conditions (*N* = 20 incident ions per pulse). The blue dashed line is included to guide the eye.

To further illustrate the dependence of *G*(–O_2_) on the irradiating ion, Figure 3 shows its variation as a function of the ion’s charge number (*Z*), from protons (*Z* = 1) to argon ions (*Z* = 18), based on our simulations conducted under identical irradiation conditions. As seen, *G*(–O_2_) increases sharply from Z = 1 to Z = 6–8, reaches a shallow maximum near Z = 8 (corresponding to ^16^O^8+^), and then gradually declines toward Z = 18. These results suggest that LET alone does not fully capture the observed trends in oxygen consumption. Instead, both LET and ion-specific track structure play a critical role in shaping radical chemistry and modulating ROD.

**Figure 4:**
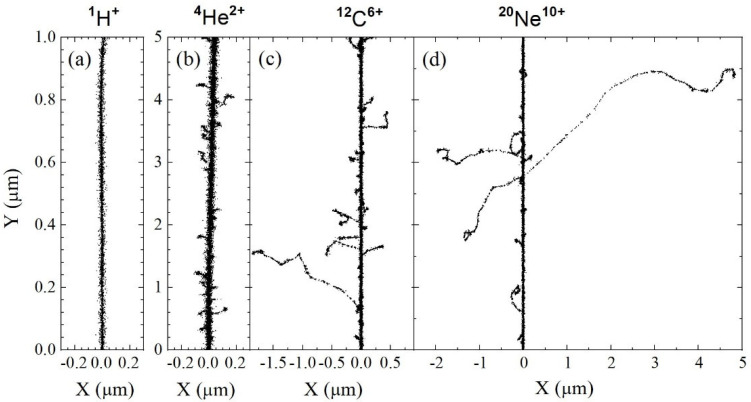
Projections onto the *XY* plane of ion track segments calculated at ~0.1 ps using our IONLYS track-structure simulation code, under identical LET conditions (~70 keV/μm) for different irradiating ions: (**a**) ^1^H^+^ (0.15 MeV), (**b**) ^4^He^2+^ (1.75 MeV/nucleon), (**c**) ^12^C^6+^ (25.5 MeV/nucleon), and (**d**) ^20^Ne^10+^ (97.5 MeV/nucleon). The ions are generated at the origin and propagate along the *Y*-axis in liquid water at 25 °C. At the incident energies considered, charge-changing collisions (i.e., electron capture and loss by the ions) are neglected. Each dot represents the spatial position of a radiolytic species formed at this time by the ionizing events, highlighting the nonhomogeneous distribution of reactive species characteristic of each ion’s track structure. Adapted from Muroya et al. [74], with permission © 2026 Radiation Research Society.

To help visualize the influence of ion-dependent track structure highlighted in Figure 2 and Figure 3, Figure 4 presents two-dimensional representations of short (1–5 μm) track segments for ^1^H^+^, ^4^He^2+^, ^12^C^6+^, and ^20^Ne^10+^ ions, calculated at ~0.1 ps under identical LET conditions (~70 keV/μm). As shown, each track exhibits a dense, continuous cylindrical “core” along the ion’s trajectory, surrounded by a more diffuse “penumbra” composed of high-energy, low-LET secondary electrons (commonly called “δ-rays”) ejected radially from the core (see, e.g., [49,71,74,75,76,77]). The extent of this penumbra increases with ion velocity, from protons to neon ions, reflecting the growing contribution of long-range δ-ray emission as ion mass and speed increase.

At fixed LET, it is precisely the increasing extent of the penumbra with ion velocity that underlies the track structure effects observed in Figure 2 and Figure 3. For protons and helium ions, the penumbra regions are very limited—virtually absent. As a result, the dominant factor influencing *G*(–O_2_) is the high local radical density within the ion trajectories due to their elevated LET (~70 keV/μm). As previously discussed, this leads to enhanced radical–radical recombination reactions, thereby reducing the availability of key radicals—primarily R^•^ and GSSG^•−^ (see Reactions (1)–(4))—to react with intracellular O_2_. Accordingly, for ^1^H^+^ and ^4^He^2+^ ions, the decrease in *G*(–O_2_) is most pronounced.

In contrast, for the heavier ions studied—carbon and neon (see Figure 4), as well as oxygen, silicon, sulfur, and argon not shown—the penumbra becomes increasingly pronounced. These extended regions arise from energetic, low-LET secondary electrons (δ-rays), ejected from the track core through knock-on collisions. δ-ray production is sporadic, and their tracks are typically well separated, resulting in a highly nonuniform spatial distribution of energy deposition. These electrons generate free radicals that remain chemically available to react with molecular oxygen, similarly to what occurs under low-LET proton irradiation. This enhanced radical availability accounts for the significantly higher *G*(–O_2_) values observed for these heavier ions compared to ^1^H^+^ and ^4^He^2+^.

However, the contribution of the penumbra appears to decline slightly beyond neon and oxygen ions. As shown in Figure 4, *G*(–O_2_) exhibits a relatively shallow maximum, which may be attributed to the increasing range of δ-rays produced by faster, heavier ions. As these high-energy electrons deposit energy over progressively larger volumes, the resulting decrease in average radical density reduces the chemical contribution of the penumbra to ROD. Under such conditions, the dense track core may once again exert greater relative influence, leading to only a modest decline in *G*(–O_2_) and accounting for the shallow maximum observed.

Taken together, these results demonstrate how increasing LET modifies the spatial pattern of energy deposition and how this, in turn, governs radical recombination dynamics and oxygen depletion. These mechanistic insights provide a foundation for interpreting ion-specific differences in radiolytic oxygen consumption, with implications for both radiation chemistry and the optimization of radiotherapeutic modalities such as FLASH and hadrontherapy.

## 4. Conclusions

This study employed our IONLYS-IRT Monte Carlo track-chemistry simulation code to examine how LET and ion-specific track structure influence radiolytic oxygen depletion in a cell-like aqueous environment under FLASH irradiation conditions. By simulating the radiolysis induced by various ion species—including protons, ^4^He^2+^, ^10^B^5+^, ^12^C^6+^, ^16^O^8+^, ^20^Ne^10+^, ^28^Si^14+^, ^32^S^16+^, and ^40^Ar^18+^—across both low and high LET regimes, we demonstrated that the oxygen consumption yield, *G*(–O_2_), depends not only on LET but also on the radiation track structure.

Our results show that *G*(–O_2_) decreases markedly with increasing LET for protons, primarily due to enhanced radical–radical recombination within the dense ionization cores of high-LET tracks. However, this LET-based trend does not fully describe the behavior of heavier ions. Under fixed LET conditions (~70 keV/μm), heavier ions such as carbon and oxygen exhibit significantly higher *G*(–O_2_) values than protons and helium ions, a discrepancy attributed to their more extended penumbra regions formed by high-energy δ-rays. These secondary electrons deposit energy over larger radial distances from the track core, producing reactive radicals that remain available to react with molecular oxygen. In contrast, the cores of lighter ions favor early-stage radical recombination, thereby limiting O_2_ consumption.

We also observed a shallow maximum in *G*(–O_2_) as a function of the ion charge number *Z*, peaking near *Z* = 8 (for ^16^O^8+^ ions), followed by a gradual decline. This feature reflects the balance between the dense ionization core and the progressively extended radical-producing penumbra associated with increasing ion charge and velocity. In other words, this trend suggests that beyond a certain ion size and velocity, the increasingly diffuse energy deposition by long-range δ-rays reduces the local radical density, thereby diminishing the chemical contribution of the penumbra to oxygen depletion. Under these conditions, the dense ionization core regains prominence in determining radiolytic chemistry and *G*(–O_2_).

Altogether, our findings underscore that LET alone is an insufficient descriptor of radiation quality in the context of radiolytic oxygen depletion. A more comprehensive understanding of radiolytic outcomes requires detailed consideration of ion-specific track structure, including the spatial heterogeneity introduced by δ-ray production. These insights have direct implications for interpreting biological responses under FLASH and hadron therapy, where oxygen availability and radical chemistry critically influence therapeutic efficacy and normal-tissue sparing.

In full agreement with the conclusions of Karle et al. [16], who underscored the potential of FLASH heavy ion therapy, our results also point to a clear therapeutic advantage for oncological hadrontherapy using protons, helium, or other heavy ions. In the tissue region preceding the tumor, ion energies are generally high and LETs are low, leading to maximal ROD and thereby enhancing the protection of surrounding healthy cells. Conversely, at the end of their path—when the ions reach the tumor volume—their energy has substantially decreased, and LET has risen sharply (Bragg peak), resulting in minimal ROD and reduced FLASH-mediated protection of tumor cells. According to our simulations, proton therapy appears particularly well suited to exploit this differential effect—maximizing both Bragg peak targeting and FLASH-mediated sparing of normal tissue. In contrast, irradiation with electrons or photons (e.g., X-rays) produces a uniform LET and consequently induces a maximal but relatively uniform FLASH effect across the entire irradiated volume.

## Data Availability

Data generated, analyzed, or supporting the findings of this study are provided in full within the article. For further inquiries, please contact the authors directly.
